# Assessing the clinical readiness of the SERENA-6 strategy

**DOI:** 10.1093/oncolo/oyaf238

**Published:** 2025-07-25

**Authors:** Rafael Balsini Barreto

**Affiliations:** Department of Clinical Oncology, Centro de Pesquisas Oncológicas (CEPON), Florianópolis, Santa Catarina, Brazil

The SERENA-6 trial presented at the plenary session of the 2025 ASCO Annual Meeting and simultaneously published in the *New England Journal of Medicine*^1^ consolidates a new therapeutic paradigm: dynamic biomarker-driven treatment switching prior to clinical progression. The authors deserve recognition for the rigorous trial design and the innovative use of *ESR1* mutation detection by circulating tumor DNA (ctDNA) to guide endocrine therapy switch in patients still deriving clinical benefit from aromatase inhibitors (AIs) and CDK4/6 inhibitors (iCDK4/6). Nonetheless, several conceptual and methodological aspects merit further consideration.

First, the use of AI maintenance in the control arm is arguably suboptimal. Several translational and clinical evidence has established that *ESR1* mutations confer resistance to AIs. Preclinical models^2^ and ctDNA-based clinical data^3^ have demonstrated that mutant estrogen receptors are constitutively active, rendering peripheral estrogen suppression ineffective. While continued AI use until radiographic progression aligns with current practice, from a molecular standpoint maintaining a therapy despite known resistance is difficult to justify.

The PADA-1 trial (NCT03079011)^4^ previously explored a similar strategy, randomizing patients receiving first-line palbociclib plus AI who developed *ESR1* mutations (in the absence of RECIST-defined progression) to either continue therapy or switch AI to fulvestrant. PADA-1 met its primary endpoint of PFS (HR 0.61; median 11.9 vs 5.7 months), suggesting that this biomarker-driven switch is feasible—even with an intramuscular SERD with less favorable pharmacokinetics and reduced activity against certain mutations.^5^

SERENA-6 builds upon this approach, utilizing the more potent oral SERD camizestrant and applying the same adaptive design, changing the treatment upon detection of *ESR1* mutation via ctDNA assay. Camizestrant has already shown activity in both pretreated and less pretreated settings, regardless of *ESR1* mutation status,^6–8^ and the current study confirms its activity, showing improvement in the primary endpoint PFS (HR 0.44; median 16.0 vs 9.2 months). Still, in the absence of mature data for more clinically relevant endpoints, it is still early to conclude that early biomarker-guided switching translates into real clinical benefit.

Time to second progression (PFS2) was also powered per protocol, although a secondary endpoint alongside overall survival (OS); however, PFS2 data are currently immature (38 events in the experimental arm and 47 in the control arm). Regardless of follow-up, I wish to highlight 3 methodological concerns:

1. **Post-progression therapy imbalance:** For PFS2 to offer meaningful insights, subsequent treatment must be predefined and balanced between arms. SERENA-6 allowed for investigator’s choice and did not permit cross-over, violating both conditions (Table 1).
2. **PFS2 origin point mismatch:** PFS2 is measured from randomization; thus, the experimental arm includes the duration on camizestrant + iCDK4/6 and subsequent therapy, while the control arm includes AI + iCDK4/6 and subsequent therapy, but it is hard to ignore that patients had already received AI + iCDK4/6 for a median of 23 months (minimum of 6 months per eligibility). Both arms subtract the months on AI before *ESR1* mutation detection, yet the experimental arm changed therapy, while the control arm remained using AI (despite a known resistance mutation). A more clinically sound approach would measure PFS2 from the start of AI + CDK4/6 until progression on camizestrant + iCDK4/6 or subsequent therapy on the control arm, with cross-over allowed and encouraged.
3. **Event definition inconsistency:** PFS2 allows both clinical and radiological progression as per investigator which could compromise the robustness of the outcome without central review.

**Table 1.** Subsequent anti-cancer regimens stratified by trial arm.

Subsequent regimen	Experimental (camizestrant)	Control (AI)
**Antibody-drug conjugate**	0	10 (12.0%)
**Chemotherapy**	26 (45.6%)	19 (22.9%)
**Oral SERD (monotherapy + combination)**	0	12 (14.4%)
**Fulvestrant (monotherapy + combination)**	11 (19.3%)	29 (34.9%)
**Other**	20 (35.1%)	13 (15.6%)

The imbalance in post-progression therapies as shown in the table complicates interpretation of OS as well, since many of the patients in the camizestrant arm received chemotherapy, while those in the AI arm received more up-to-date treatments—with survival gains over chemotherapy—such as antibody-drug conjugates and endocrine combinations. As the OS data mature, it will be important to interpret them in the context of such post-progression differences. Such variability on subsequent therapy may also impact quality-of-life (QoL) outcomes—considered a hard endpoint by health agencies and an exploratory outcome per the SERENA-6 protocol. Earlier disease progression certainly affects QoL negatively, but so does early exposure to more toxic therapies. This imbalance may confound QoL assessments.

SERENA-6 appears to consolidate existing knowledge rather than introduce new concepts: it confirms the efficacy of camizestrant in patients with ESR1 mutations detected via ctDNA; demonstrates superiority over AIs in a setting of established molecular resistance; and reinforces the biomarker-guided treatment switch strategy previously proposed in the PADA-1 study, implemented prior to clinical progression, with a median PFS gain of approximately 6 months comparable to that observed with fulvestrant in this context.

The SERENA-4 trial (NCT04711252),^9^ which compares camizestrant to anastrozole in combination with palbociclib as first-line treatment, presents a more appropriate design from a regulatory standpoint. By employing a standard-of-care comparator arm, it may clarify whether initiating therapy with a novel oral SERD—thereby anticipating the main mechanism of resistance—will offer clinical benefit. This rationale mirrors that of the FLAURA study (NCT02296125), in which osimertinib demonstrated first-line superiority by preemptively targeting the T790M resistance mutation.^10^

SERENA-6 met its primary endpoint, but relying solely on PFS and PFS2 is misleading in a setting such as metastatic hormone-positive breast cancer where patients receive multiple sequential therapies. With OS data immature, no cross-over and no standardized post-progression treatment, it remains uncertain whether early change of therapy leads to meaningful long-term benefit. Widespread implementation of the SERENA-6 strategy also poses logistical and economic challenges: real-time *ESR1* mutation monitoring via ctDNA every 2-3 months demands substantial technical and financial resources—often beyond the reach of public systems or private insurers. We risk adopting a new standard based on a biologically compelling rationale yet without robust evidence of improved survival or QoL outcomes.
